# Analysis of the CHS Gene Family Reveals Its Functional Responses to Hormones, Salinity, and Drought Stress in Moso Bamboo (*Phyllostachys edulis*)

**DOI:** 10.3390/plants14020161

**Published:** 2025-01-08

**Authors:** Shiying Su, Xueyun Xuan, Jiaqi Tan, Zhen Yu, Yang Jiao, Zhijun Zhang, Muthusamy Ramakrishnan

**Affiliations:** 1 State Key Laboratory of Subtropical Silviculture, Bamboo Industry Institute, Zhejiang A&F University, Lin’an, Hangzhou 311300, China; sushiying@stu.zafu.edu.cn (S.S.); xuanxueyun@126.com (X.X.); tan@stu.zafu.edu.cn (J.T.); yuq82733@gmail.com (Z.Y.); jiaoyang19980227@163.com (Y.J.); 2 State Key Laboratory of Tree Genetics and Breeding, Co-Innovation Center for Sustainable Forestry in Southern China, Bamboo Research Institute, Key Laboratory of National Forestry and Grassland Administration on Subtropical Forest Biodiversity Conservation, School of Life Sciences, Nanjing Forestry University, Nanjing 210037, China

**Keywords:** *Phyllostachys edulis*, *CHS* gene family, transcription factors, hormones, abiotic stress

## Abstract

Chalcone synthase (CHS), the first key structural enzyme in the flavonoid biosynthesis pathway, plays a crucial role in regulating plant responses to abiotic stresses and hormone signaling. However, its molecular functions remain largely unknown in *Phyllostachys edulis*, which is one of the most economically and ecologically important bamboo species and the most widely distributed one in China. This study identified 17 *CHS* genes in *Phyllostachys edulis* and classified them into seven subgroups, showing a closer evolutionary relationship to *CHS* genes from rice. Further analysis of *PeCHS* genes across nine scaffolds revealed that most expansion occurred through tandem duplications. Collinearity analysis indicated strong evolutionary conservation among *CHS* genes. Motif and gene structure analyses confirmed high structural similarity, suggesting shared functional characteristics. Additionally, *cis*-acting element analysis demonstrated that *PeCHS* genes are involved in hormonal regulation and abiotic stress responses. RNA-Seq expression profiles in different bamboo shoot tissues and heights, under various hormone treatments (gibberellin (GA), naphthaleneacetic acid (NAA), abscisic acid (ABA), and salicylic acid (SA)), as well as salinity and drought stress, revealed diverse response patterns among *PeCHS* genes, with significant differential expression, particularly under hormone treatments. Notably, *PeCHS14* consistently maintained high expression levels, suggesting its key role in stress response mechanisms. qRT-PCR analysis further validated the expression differences in five *PeCHS* genes under GA and ABA treatments. Subcellular localization analysis demonstrated that PeCHS14 and PeCHS15 proteins are localized in the nucleus. This study provides a foundation for investigating the potential functions of *PeCHS* genes and identifies candidate genes for future research on the responses of *Phyllostachys edulis* to abiotic stresses and hormone signaling.

## 1. Introduction

Chalcone synthase (CHS) in plants belongs to the type III polyketide synthase (PKS) supergene family [1], which also includes stilbene synthase (STS) [2], curcuminoid synthase (CUS) [3], and alkylresorcylic acid synthase (ARAS) [4]. Studies have demonstrated that members of the PKS superfamily play critical roles in resisting biotic and abiotic stresses and promoting the synthesis of secondary metabolites such as anthocyanins and flavonoids [5,6,7,8,9,10]. CHS is the most well-studied representative of this superfamily and is believed to have existed and diversified as early as gymnosperms [11]. Most plants contain at least one *CHS* gene. For instance, 27 *CHS* members have been identified in rice (*Oryza sativa*) [12], 14 in maize (*Zea mays*) [13], and 5 in mulberry (*Morus atropurpurea* Roxb.) [14], as well as additional members in cassava (*Manihot esculenta* Crantz.) [15] and *Kochia scoparia* [16]. Chalcone synthase (CHS) is the first key structural enzyme in the flavonoid biosynthesis pathway [17]. It catalyzes the condensation of one molecule of *p*-coumaroyl-CoA with three molecules of malonyl-CoA to form naringenin chalcone [18,19,20].

As a precursor to various flavonoids, naringenin chalcone undergoes further enzymatic reactions to produce structurally diverse and physiologically active polyketide compounds, thereby enhancing flavonoid biosynthesis. Flavonoids, including chalcones, flavanones, flavones, and anthocyanins [17], play critical roles in abiotic stress resistance, such as UV radiation and drought tolerance [21,22] and influencing auxin transport [23]. Additionally, numerous studies suggest that the *CHS* gene family facilitates anthocyanin synthesis [24], enhances UV radiation resistance [25,26,27,28], and participates in the salicylic acid (SA) pathway [29], thus playing essential roles in responding to both biotic and abiotic stresses [30,31]. For instance, in apple (*Malus domestica*), the upregulation of *CHS* genes increases anthocyanin content, mitigating oxidative damage under drought stress [32].

In *Oryza sativa*, RNA-Seq expression profiling under abiotic stress and hormone treatments revealed the transcriptional activity of 18 *OsCHS* genes in response to abscisic acid (ABA) treatment [12]. In tobacco (*Nicotiana tabacum*), CHS family members *NtCHS1* and *NtCHS3* showed significantly increased expression under drought and salinity stress, as well as treatments with 2,4-D, ABA, and MeJA hormones [13]. In sweet cherry (*Prunus avium* L.), *CHS* genes contributed to improved germination rates and seedling fresh weight under drought stress [33]. The overexpression of *EaCHS1* in *Eupatorium adenophorum* enhanced salinity stress resistance during seed germination and root development [34]. Furthermore, the increased expression of the *PdCHS3* gene in pear (*Pyrus betulaefolia*) effectively boosted resistance to black spot disease [35].

Moso bamboo *(Phyllostachys edulis),* a member of the Poaceae family and Bambusoideae subfamily, is one of the most widely distributed and economically significant bamboo species in China, valued for its high ecological and economic importance [36]. As a globally important dual-purpose resource for shoots and timber, various parts of *Phyllostachys edulis* contain multiple flavonoid compounds with antioxidant, free radical-scavenging, and anticancer activities [37,38], making it widely used in the medicine [39] and food industries [40]. However, during growth and development, *Phyllostachys edulis* frequently encounters abiotic stresses such as drought, low temperatures, high temperatures, and salinity, which hinder its growth and development [41].

In this study, 17 *CHS* gene family members were identified in *Phyllostachys edulis* through bioinformatics analyses. Comprehensive analyses were conducted, including physicochemical property assessments, phylogenetic relationships, chromosome localization, intra- and interspecies collinearity, motifs and gene structures, *cis*-acting elements, Gene Ontology (GO) functional enrichment, and transcription factor regulatory networks. Combining RNA-Seq expression profiles and qRT-PCR results, the response mechanisms of the *CHS* gene family in *Phyllostachys edulis* to abiotic and hormonal stresses were elucidated. Subcellular localization analyses demonstrated that PeCHS14 and PeCHS15 proteins are localized in the nucleus. This study provides a foundation for further investigations into the stress resistance functions of the *CHS* gene family in *Phyllostachys edulis* and identifies candidate genes for studying its stress resistance mechanisms.

## 2. Results

### 2.1. Identification and Physicochemical Property Analysis of the PeCHS Gene Family Members

Using the Pfam models of plant CHS domains (PF00195 and PF02797), an HMMER3 search was performed on *Phyllostachys edulis* protein database with an E-value threshold of ≤10^−20^. A total of 23 putative *CHS* candidate genes were identified. After removing redundant sequences and verifying conserved domains and motifs, 17 *CHS* gene family members were ultimately identified (Table 1) and designated as *PeCHS01* to *PeCHS17* based on their chromosomal positions.

The proteins encoded by these 17 *CHS* genes ranged in length from 309 amino acids (PeCHS12) to 405 amino acids (PeCHS01), with molecular weights (MWs) between 32.996 kDa (PeCHS12) and 43.789 kDa (PeCHS09). Predicted isoelectric points (pI) varied from 5.54 (PeCHS15) to 7.08 (PeCHS02). Stability analysis indicated that 12 CHS proteins (71%) had instability indices below 40, classifying them as stable proteins, while PeCHS02, PeCHS06, PeCHS10, PeCHS14, and PeCHS16 had instability indices above 40, suggesting that they are unstable in vitro. The grand average of hydropathicity (GRAVY) scores ranged from −0.096 (PeCHS10) to 0.079 (PeCHS12). Notably, 12 CHS proteins (71%) had negative GRAVY values, indicating that PeCHS proteins are predominantly hydrophilic.

### 2.2. Classification and Phylogenetic Analysis of the PeCHS Gene Family

**Figure 1:**
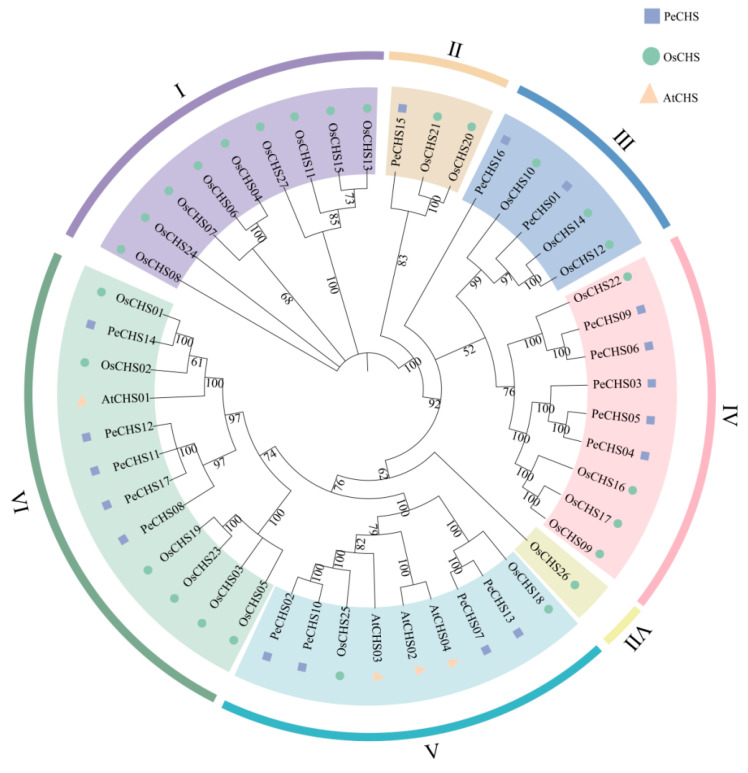
Phylogenetic analysis of interspecies relationships of PeCHS. The phylogenetic tree was constructed using MEGA 7.0 with the Neighbor-Joining (NJ) method. The evolutionary relationships among CHS sequences from *Phyllostachys edulis* (purple squares), *Arabidopsis thaliana* (orange triangles), and *Oryza sativa* (green circles) are illustrated. AtCHS represents CHS sequences from *Arabidopsis thaliana*, while OsCHS represents CHS sequences from *Oryza sativa*.

To elucidate the evolutionary relationships between PeCHS proteins and CHS proteins from other species, a phylogenetic tree was constructed using the amino acid sequences of 17 CHS proteins from *Phyllostachys edulis*, 27 from rice, and 4 from *Arabidopsis thaliana* (Figure 1). The results revealed that the *CHS* gene family could be divided into seven classes. Among these, Class IV and Class VI contained the largest proportion of PeCHS members, with five members each, accounting for 29.4% of the total. Class V followed with four members (23.5%), while Class III contained two members (11.8%), and Class II included one member (5.9%). Notably, no PeCHS members were identified in Class I or Class VII. As a dicotyledonous species, *Arabidopsis* exhibited an evolutionary relationship distinct from *Phyllostachys edulis* and *Oryza sativa*. Compared with *Arabidopsis thaliana*, the PeCHS proteins displayed a closer phylogenetic relationship to the CHS proteins from rice, reflecting the stronger evolutionary ties and adaptations among monocot species.

### 2.3. Chromosomal Localization and Collinearity Analysis of PeCHS Genes

**Figure 2:**
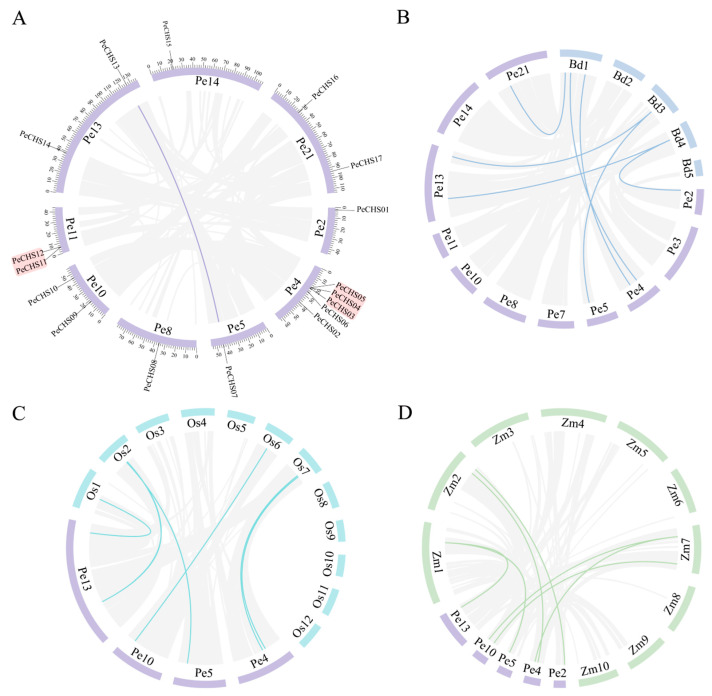
Intraspecies and interspecies collinearity analysis of *PeCHS* genes. (**A**) Chromosomal distribution and interchromosomal relationships of *PeCHS* genes. (**B**) Interspecies collinearity analysis of CHS genes between *Phyllostachys edulis* and *Brachypodium distachyon.* (**C**) Interspecies collinearity analysis of *CHS* genes between *Phyllostachys edulis* and *Oryza sativa.* (**D**) Interspecies collinearity analysis of *CHS* genes between *Phyllostachys edulis* and *Zea mays*. Pe represents the chromosomes of *Phyllostachys edulis*, Bd represents the chromosomes of *Brachypodium distachyon*, Os represents the chromosomes of *Oryza sativa*, and Zm represents the chromosomes of *Zea mays*.

Using TBtools, the 17 *PeCHS* genes were mapped to their respective positions on *Phyllostachys edulis* scaffolds (Figure 2A), and their chromosomal distribution was analyzed. The *PeCHS* genes were unevenly distributed across nine scaffolds. Among these, scaffold 4 contained the highest number of *PeCHS* genes, with a total of five. Scaffolds 10, 11, 13, and 21 each contained two *PeCHS* genes, while scaffolds 2, 5, 8, and 14 each harbored one *PeCHS* gene. Gene duplication events, which play a vital role in generating new genes and regulatory pathways, are crucial for plant genome variation. Intraspecies collinearity analysis (Figure 2A) revealed that only one gene pair within the *PeCHS* gene family arose through segmental duplication, suggesting a relatively conserved evolutionary pattern. Furthermore, gene clusters were identified on scaffolds 4 and 11, likely resulting from tandem duplication events. These findings indicate that the expansion of the *PeCHS* gene family in *Phyllostachys edulis* was primarily driven by tandem duplication, reflecting its evolutionary conservation.

To further investigate the evolutionary origins of *PeCHS* genes, a collinearity analysis was performed using the genomes of the Poaceae model plants *Brachypodium distachyon*, *Oryza sativa*, and *Zea mays* (Figure 2B–D). The results revealed seven orthologous gene pairs between *Phyllostachys edulis* and both *Brachypodium distachyon* and *Zea mays*, and six orthologous gene pairs between *Phyllostachys edulis* and *Oryza sativa.* These findings suggest a conserved evolutionary relationship between *PeCHS* genes and their counterparts in other Poaceae species, providing insights into the functional and evolutionary significance of this gene family.

### 2.4. Motif and Gene Structure Analysis of the PeCHS Gene Family

**Figure 3:**
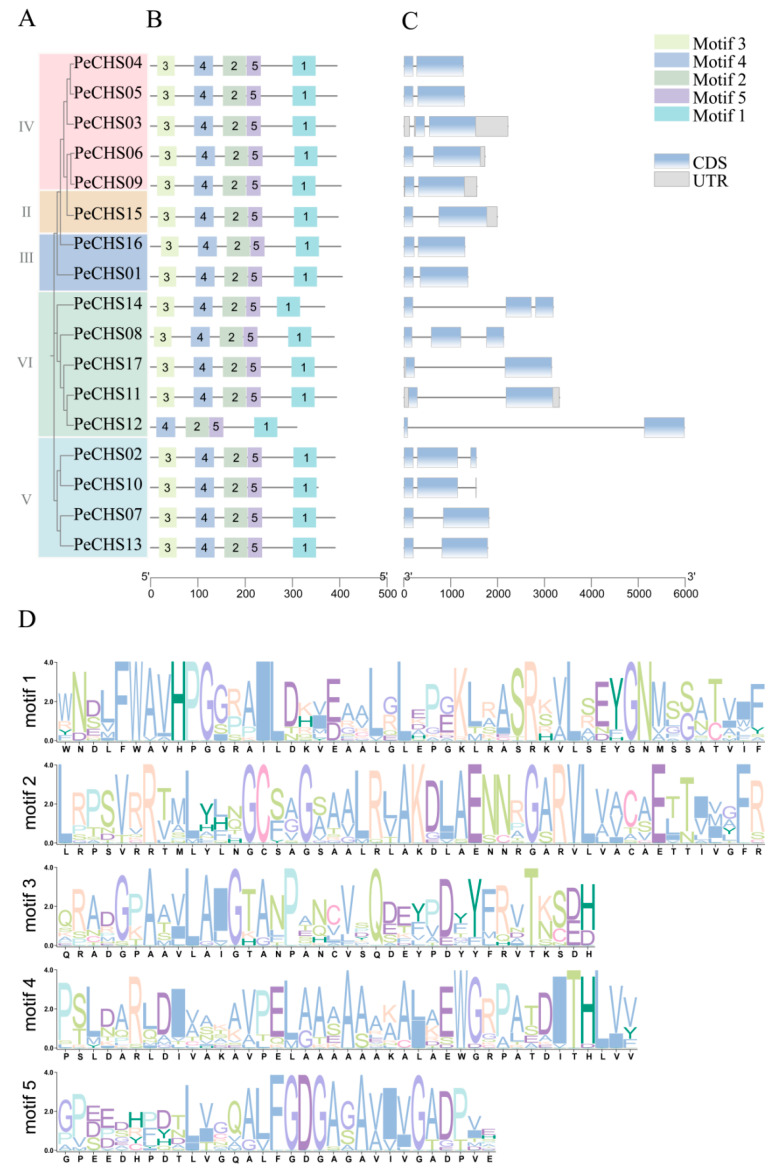
Phylogenetic relationships, conserved motifs, and gene structures of *PeCHS* genes. (**A**) A Neighbor-Joining (NJ) phylogenetic tree of PeCHS proteins constructed using MEGA. (**B**) Conserved motifs in PeCHS proteins, with colored boxes representing motifs 1–5. (**C**) Gene structures of *PeCHS* genes, showing introns (gray lines), exons (blue rectangles), and untranslated regions (UTRs, gray rectangles). (**D**) Motif logo displaying the conserved sequences and relative frequencies of motifs in PeCHS proteins.

Motif and gene structure investigations were conducted to enhance the understanding of the categorization and structural makeup of the *PeCHS* gene family. According to conserved motifs and domains in the PeCHS protein sequences, the gene family was classified into five categories (Figure 3A), aligning with the findings of the phylogenetic tree analysis. Motif analysis (Figure 3B) revealed that the majority of *PeCHS* gene family members possessed a complete motif structure. All sequences, with the exception of PeCHS12, which was devoid of motif 3, contained the five conserved motifs. Gene structure analysis (Figure 3C) revealed high structural similarity among *PeCHS* genes. The majority of the genes (12, 71%) contained one intron and two exons, while a smaller proportion (5, 29%) had two introns and three exons. Closely related *PeCHS* genes in the phylogenetic tree exhibited similar structures. For instance, *PeCHS01* and *PeCHS16*, both belonging to Class III, contained one intron and two exons of identical lengths. Additionally, motif logo analysis (Figure 3D) indicated that the PeCHS motifs were highly conserved. The five members of Class VI had relatively longer introns, suggesting that this clade might have experienced distinct selective pressures during evolution, potentially leading to functional divergence.

### 2.5. Conserved Domain and Tertiary Structure Prediction of PeCHS Proteins

**Figure 4:**
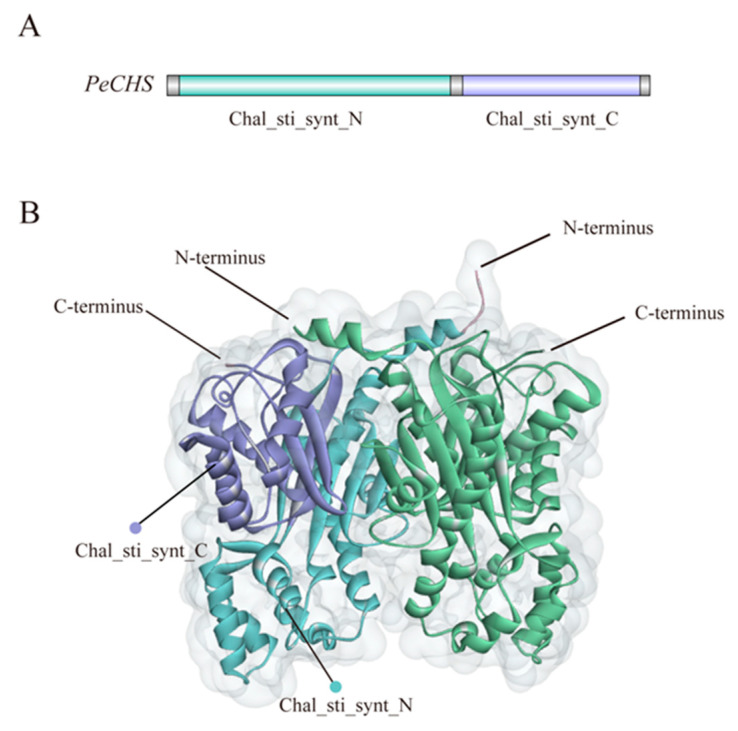
Conserved domains and tertiary structure prediction of PeCHS proteins. (**A**) Conserved domains. Blue represents the Chal_sti_synt_N domain, and purple represents the Chal_sti_synt_C domain. (**B**) Tertiary structure.

The *PeCHS* gene family contains two conserved domains: Chal_sti_synt_N and Chal_sti_synt_C (Figure 4A). Tertiary structure predictions (Figure 4B) indicate that PeCHS proteins primarily form tertiary structures composed of two subunits functioning as homodimers. The N-terminal α-helices of the two monomers intertwine to form a stable dimerization interface. This structural arrangement aligns with the functional characteristics of CHS proteins and highlights their importance in catalytic activity and substrate specificity.

### 2.6. Cis-Acting Element Analysis of the PeCHS Gene Family

*Cis*-acting elements are unique DNA sequences situated upstream of structural genes that serve as binding sites for transcription factors. By interacting with transcription factors, these elements regulate the initiation and efficiency of gene transcription, playing a critical role in plant stress responses and growth regulation.

**Figure 5:**
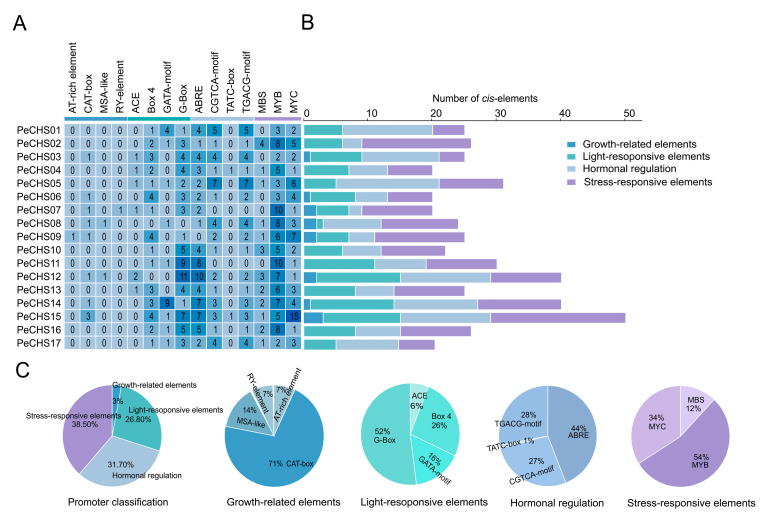
*Cis*-regulatory elements in the promoter region (upstream 2000 bp) of *PeCHS* genes. (**A**) Different types of *cis*-regulatory elements upstream of *PeCHS* genes in *Phyllostachys edulis*, with the numbers inside the boxes representing the count of each element. (**B**,**C**) The number and proportion of different types of *cis*-regulatory elements upstream of *PeCHS* genes.

The analysis of the 2000 bp upstream regions of *PeCHS* genes identified four categories of *cis*-acting elements (Figure 5): growth and development-related elements (14, 3%), light-responsive elements (126, 26.8%), hormone regulation elements (149, 31.7%), and stress-responsive elements (181, 38.5%). Among these, stress-responsive and hormone regulation elements were the most abundant. All *PeCHS* genes contained MYB and MYC elements, with 16 (94.12%) also harboring ABRE and G-box elements. These findings suggest that these *cis*-acting elements play essential roles in the response of *Phyllostachys edulis* to abiotic and hormonal stresses.

### 2.7. RNA-Seq Expression Profile Analysis of the PeCHS Gene Family

**Figure 6:**
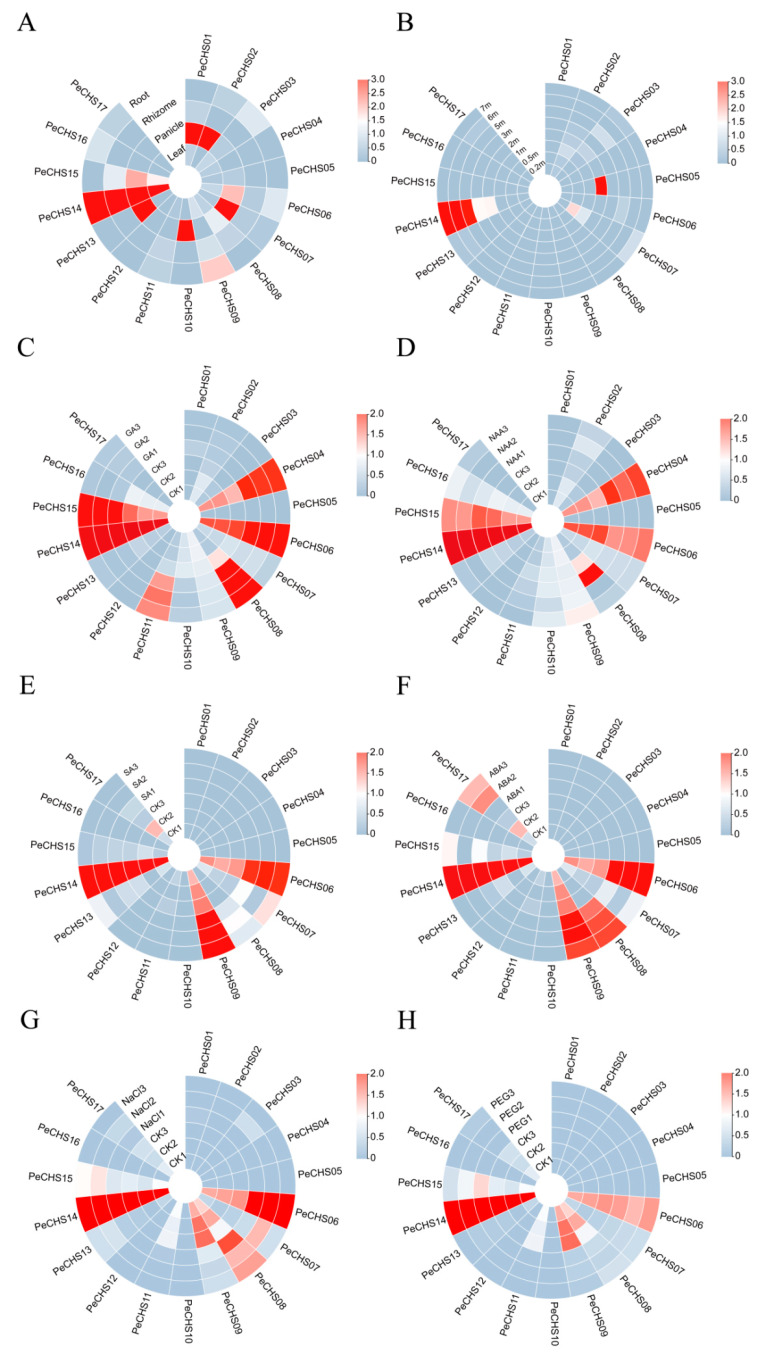
Heatmap of *PeCHS* gene expression (log2^(TPM+1)^) in *Phyllostachys edulis*: (**A**) Expression in roots, stems, panicle inflorescences, and leaves. (**B**) Expression in shoots at different heights. (**C**) Expression under GA treatment. (**D**) Expression under NAA treatment. (**E**) Expression under SA treatment. (**F**) Expression under ABA treatment. (**G**) Expression under NaCl treatment. (**H**) Expression under PEG treatment. The relative expression levels are represented by a color scale, with blue indicating low expression and red indicating high expression.

Based on published RNA-Seq data, the expression patterns of the *PeCHS* gene family across different tissues were analyzed in greater detail. The results indicated that the *CHS* gene family in *Phyllostachys edulis* displayed differential expression patterns across tissues (Figure 6A). *PeCHS01*, *PeCHS02*, *PeCHS07*, *PeCHS10*, *PeCHS13*, and *PeCHS14* showed elevated expression levels in inflorescences, with *PeCHS14* maintaining consistently high levels across all tissues. An analysis of gene expression profiles in bamboo shoots of different heights (Figure 6B) revealed that most *PeCHS* genes had low expression levels irrespective of shoot height. However, *PeCHS14* showed a unique expression pattern, with its expression levels increasing progressively as the shoot grew beyond 2 m. *PeCHS05* exhibited peak expression at a height of 2 m but demonstrated low expression levels at other growth stages.

In hormone treatment experiments, the *PeCHS* gene exhibited differential responses to gibberellin (GA) treatment relative to the control group (Figure 6C). The expression levels of *PeCHS04*, *PeCHS06*, *PeCHS08*, *PeCHS11*, and *PeCHS15* were upregulated under GA treatment, whereas *PeCHS14* consistently maintained high expression levels with no significant changes. Under naphthaleneacetic acid (NAA) treatment (Figure 6D), *PeCHS04* exhibited an overall upregulation pattern, while *PeCHS06* and *PeCHS08* showed consistent downregulation trends. Under salicylic acid (SA) treatment (Figure 6E), *PeCHS06*, *PeCHS07*, *PeCHS08*, and *PeCHS09* consistently exhibited upregulation patterns. Similarly, under abscisic acid (ABA) treatment (Figure 6F), these four genes also exhibited overall upregulation patterns. These findings indicate that specific *PeCHS* genes may be involved in regulating plant signal transduction or metabolic pathways in response to distinct hormone stimuli.

Under NaCl treatment (Figure 6G), the expression level of *PeCHS06* was markedly upregulated relative to the control group, whereas *PeCHS09* and *PeCHS11* exhibited downregulation trends. Under PEG treatment (Figure 6H), most PeCHS genes, including *PeCHS08*, *PeCHS09*, *PeCHS11*, and *PeCHS17*, exhibited reduced expression levels and consistent downregulation trends. Additionally, *PeCHS14* consistently maintained elevated expression levels under both NaCl and PEG conditions.

### 2.8. GO Functional Enrichment Analysis of the PeCHS Gene Family

**Figure 7:**
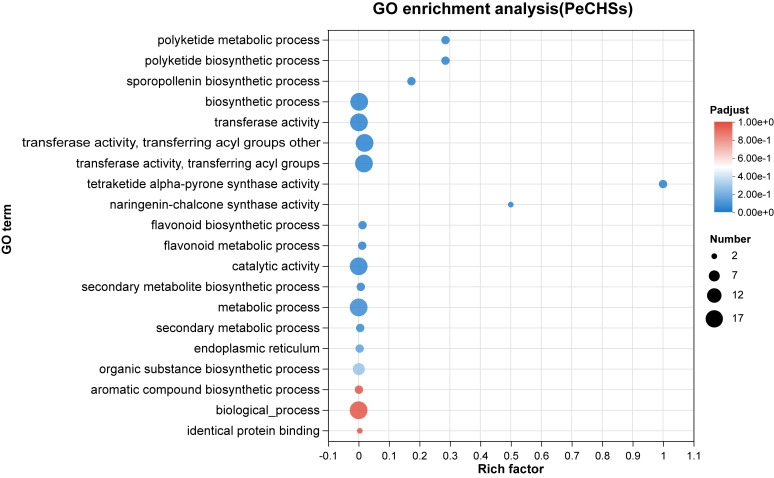
Top 20 most enriched GO terms for *PeCHS* genes. The horizontal axis represents the enrichment factor, and the size of the circles indicates the number of genes annotated with the given GO term.

To predict the biological functions of the *CHS* gene family in *Phyllostachys edulis*, GO annotation and enrichment analysis were performed on 17 *PeCHS* genes. The results of the GO enrichment analysis (Figure 7) revealed that the functions of the *PeCHS* gene family were primarily enriched in “tetraketide αlpha-pyrone synthase activity”, “naringenin-chalcone synthase activity”, “polyketide biosynthetic process”, “polyketide metabolic process”, and “sporopollenin biosynthetic process”. Among these, “tetraketide αlpha-pyrone synthase activity” had the highest enrichment factor, followed by “naringenin-chalcone synthase activity”. Furthermore, “activity” and “biosynthesis” appeared most frequently within the functional modules, indicating that *PeCHS* genes mainly participate in functions related to bioactivity and the synthesis of chemical compounds within *Phyllostachys edulis*.

### 2.9. Correlation Analysis of the Regulatory Network Between PeCHS Genes and Transcription Factors

**Figure 8:**
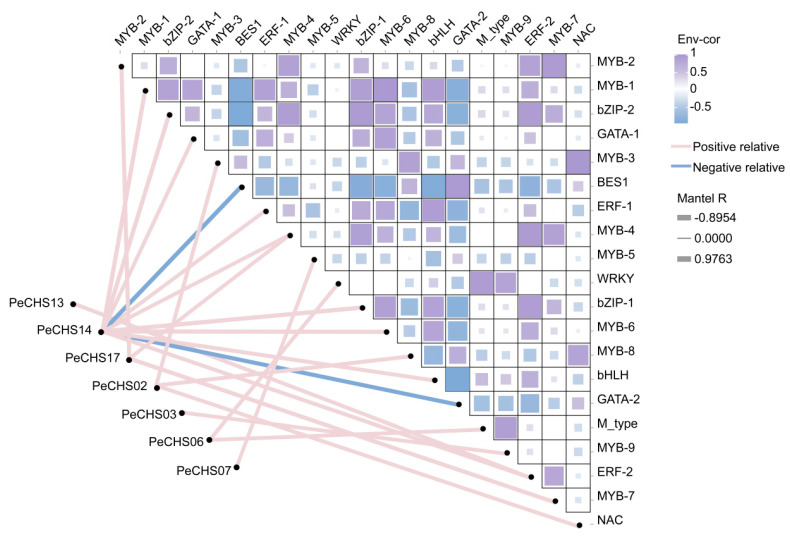
Correlation between *PeCHS* genes and transcription factor genes. The edge width of the lines represents the corresponding correlation strength, and the line color indicates statistical significance.

By constructing a dynamic network heatmap, the correlations between *PeCHS* genes and their regulatory transcription factors were analyzed in a more intuitive manner. The results (Figure 8) revealed that *PeCHS02*, *PeCHS03*, *PeCHS06*, *PeCHS07*, *PeCHS13*, *PeCHS14*, and *PeCHS17* occupied central positions in the network and were predominantly regulated positively by transcription factors such as MYB and bZIP. Furthermore, most transcription factors were positively correlated with *PeCHS14*, whereas BES1 and GATA-2 exhibited negative correlations with *PeCHS14*, potentially exerting inhibitory effects on its expression.

### 2.10. Subcellular Localization of PeCHS14 and PeCHS15

**Figure 9:**
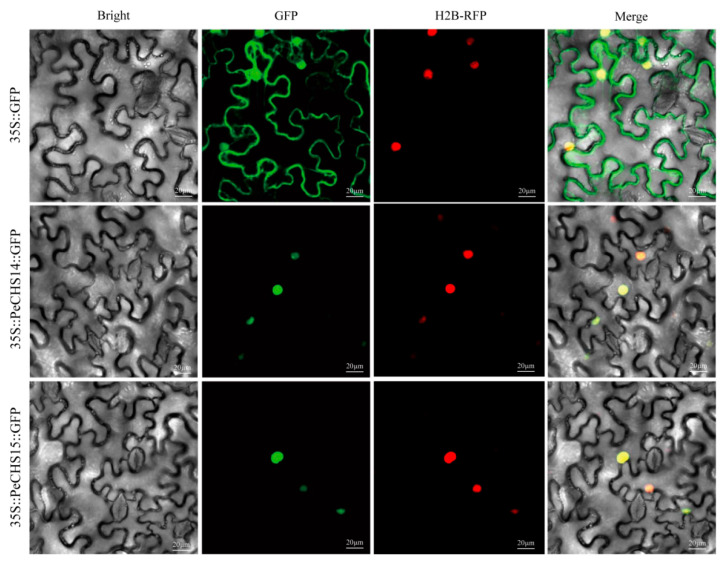
Subcellular localization of GFP-fused PeCHS14 and PeCHS15 proteins. Bright: Bright-field image of *Nicotiana benthamiana* epidermal cells. GFP: Green fluorescence signal emitted by GFP. H2B-RFP: Red fluorescence signal emitted by nuclear H2B-RFP. Merge: Overlaid image of the two fluorescence signals, with yellow fluorescence indicating the overlap of green and red signals. Bars = 20 μm.

To further investigate the subcellular localization of PeCHS proteins in *Phyllostachys edulis*, PeCHS14 and PeCHS15 were selected for analysis. A transient expression system based on *Nicotiana benthamiana* epidermal cells was utilized to analyze their subcellular localization. The intracellular distribution of PeCHS14 and PeCHS15 was confirmed by detecting green fluorescence (GFP) and red fluorescence (H2B-RFP) signals (Figure 9). The results indicated that GFP signals were predominantly concentrated in the nuclear region, overlapping significantly with nuclear signals marked by H2B-RFP. The merged images displayed distinct yellow fluorescence, further verifying that PeCHS14 and PeCHS15 are primarily localized in the nucleus.

### 2.11. QRT-PCR Analysis of the PeCHS Gene Family

**Figure 10:**
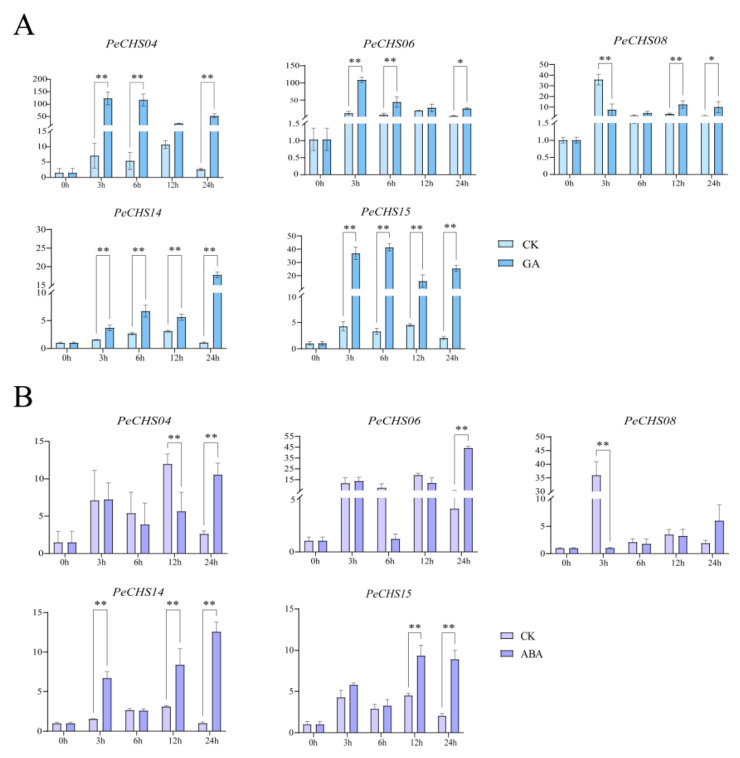
Expression patterns of the *PeCHS* gene family under hormone treatments. (**A**) Expression patterns of the *PeCHS* gene family under GA treatment. (**B**) Expression patterns of the *PeCHS* gene family under ABA treatment. (*: *p* ≤ 0.05; **: *p* ≤ 0.01).

To confirm the reliability of RNA-Seq data, qRT-PCR was conducted to analyze the expression patterns of five *PeCHS* gene family members (*PeCHS04*, *PeCHS06*, *PeCHS08*, *PeCHS14*, and *PeCHS15*) under GA and ABA treatments at 0 h, 3 h, 6 h, 12 h, and 24 h. The results indicated that under GA treatment (Figure 10A), the expression levels of the class IV subfamily genes *PeCHS04* and *PeCHS06* were significantly upregulated compared to the control group, peaking at 3 h, with levels approximately 8-fold and 60-fold higher than those in the untreated group, respectively. In contrast, the class VI subfamily genes *PeCHS08* and *PeCHS14* exhibited distinct response patterns: *PeCHS08* was significantly downregulated at 3 h but gradually upregulated at later time points, whereas *PeCHS14* was continuously upregulated throughout the GA treatment, peaking at 24 h. *PeCHS15* exhibited an upregulation trend across all GA treatment time points.

Under ABA treatment (Figure 10B), the five genes exhibited diverse expression patterns. Compared to the control group, *PeCHS04* and *PeCHS06* showed a slight upregulation at 3 h, followed by a downregulation trend, and then a re-upregulation at 24 h, with *PeCHS06* showing an approximately 8-fold increase compared to the control group at 24 h. *PeCHS08* was significantly downregulated at 3 h, 6 h, and 12 h of ABA treatment, but began to upregulate at 24 h. *PeCHS14* exhibited a slight downregulation at 6 h, with upregulation at other time points, reaching its highest expression level at 24 h. *PeCHS15* demonstrated an overall upregulation trend under ABA treatment; however, its expression pattern was unstable, showing fluctuations with upregulation at 3 h, downregulation at 6 h, a subsequent increase at 12 h, and a decline again at 24 h.

## 3. Discussion

To investigate the functions and evolutionary relationships of the *PeCHS* gene family, this study identified 17 members. Phylogenetic analysis classified the *PeCHS* gene family into seven distinct groups. Gene structure analysis revealed that the *PeCHS* gene family contains relatively few introns, with most members having one intron and a few possessing two (Figure 3C). Gene families with fewer introns tend to evolve relatively late, experience greater environmental pressures, and exhibit limited or specialized functions. Such gene families are also more likely to be induced by external stresses [42]. Hence, the *PeCHS* gene family is speculated to play a significant role in plant responses to abiotic and biotic stresses. Furthermore, Liu et al. reported that extensive gene duplication drives the expansion of intron-poor gene families [42]. Notably, intraspecies collinearity analysis identified two gene clusters located on scaffold 4 and scaffold 11 (Figure 2A). Motif analysis indicated that, except for PeCHS12, which lacks motif 3, most *PeCHS* gene family members contain five conserved motifs (Figure 3B). Taken together, these findings suggest that the *PeCHS* gene family has undergone relatively conserved evolutionary processes. It is hypothesized that the *PeCHS* gene family underwent whole-genome duplication (WGD) events, resulting in gene duplication, loss, and functional differentiation during evolution.

*Cis*-acting elements regulate gene expression through interactions with transcription factors or other DNA-binding proteins, playing vital roles in plant stress responses and growth regulation. An analysis of *cis*-acting elements in the *PeCHS* gene family revealed that most genes harbor stress-responsive, light-responsive, and hormone-regulatory elements (Figure 5), with stress-responsive and hormone-regulatory elements being predominant. Among the stress-responsive elements, MYB elements interact with MYB transcription factors and are activated under abiotic stress and exogenous ABA treatments [43]. Similarly, MYC elements and their transcription factors are involved in drought resistance [44], salinity stress [45], and the regulation of ABA-responsive gene expression [46]. All *PeCHS* gene members were identified to contain MYB and MYC elements (Figure 5A), suggesting that these elements play crucial roles in the stress and hormone responses of *PeCHS* genes. Additionally, most *PeCHS* genes also harbor ABRE and G-box elements. ABRE elements mediate the expression of stress-responsive genes in an ABA-dependent manner, thereby enhancing resistance to drought, high salinity, low temperatures, and ABA [47]. G-box elements, as light-responsive components, are ubiquitous in plants and interact with other ABA-responsive complexes to mediate ABA responses [48]. These findings suggest that *PeCHS* gene family members are closely linked to ABA regulation and expression, as supported by the RNA-Seq expression profiling (Figure 6F) and qRT-PCR results (Figure 10B). *Phyllostachys edulis* is well known for its rapid growth, a feature that has been extensively studied. Studies indicate that GA is a key hormone involved in triggering bamboo’s rapid growth [49]. RNA-Seq expression profiling (Figure 6C) revealed the significant upregulation of five *PeCHS* genes under GA treatment, consistent with the qRT-PCR results (Figure 10A). This suggests that these five *PeCHS* genes are sensitive to GA and likely play pivotal roles in GA-mediated signaling pathways and the rapid growth mechanism of bamboo.

Transcription factors are key regulators of gene expression, orchestrating the activity of multiple functional genes and profoundly influencing the biosynthesis of secondary metabolites in plants [50]. Under stress conditions, transcription factors activate specific genes and facilitate protein synthesis [51]. Various types of transcription factors, including WRKY, bZIP, MYB, and NAC, play pivotal roles in plants responding to stress [52,53,54,55,56]. Further research indicates that transcription factors regulating *CHS* gene expression predominantly belong to the MYB, bHLH, and WD40 families [57,58]. These three transcription factor families form the MBW complex, which facilitates flavonoid accumulation, including anthocyanins, supports plant development, and bolsters resistance to biotic and abiotic stresses [59,60]. Among these, MYB transcription factors enhance the expression of target genes, safeguard plants under stress, and drive the biosynthesis of secondary metabolites such as flavanols, anthocyanins, and flavonoids [61,62,63,64,65,66]. For instance, *GMYB10* enhances anthocyanin synthesis in colored gerbera leaves under stress conditions [67]. *MsMYB741* increases the expression of *MsPAL1* and *MsCHI*, encouraging flavonoid accumulation and root exudation to improve aluminum resistance in alfalfa [68]. In blueberries, *VcMYB* plays a central role in regulating anthocyanin synthesis within the ABA signaling pathway [69].

In this study, the correlation analysis of the transcription factor regulatory network indicated that most *PeCHS* genes are positively regulated by MYB transcription factors (Figure 8), suggesting that these transcription factors may facilitate flavonoid biosynthesis and responses to abiotic stress and hormone treatments in the *PeCHS* gene family. Notably, further analysis revealed a negative correlation between *PeCHS14* and the BES1 and GATA-2 transcription factors. BES transcription factors are known to positively regulate BR signaling pathways and responses to abiotic stresses such as drought, high temperatures, and salinity stress. For instance, *OsBZR1* plays a vital role in BR signaling transduction in *Oryza sativa* [70]. BES transcription factors in wheat (*Triticum aestivum*) significantly enhance drought tolerance in transgenic plants [71], while *SlBZR1D* in tomato positively regulates salinity tolerance by upregulating several stress-related genes [72]. GATA transcription factors are closely related to photosynthesis and growth development. For example, *OsGATA16* in rice positively regulates chlorophyll biosynthesis and chloroplast development [73], whereas *PeGATA26* in bamboo significantly inhibits primary root length and plant height in transgenic *Arabidopsis thaliana* [74]. These findings suggest that the expression of *PeCHS14* may also be influenced by these two transcription factors, although the exact regulatory mechanisms require further experimental validation.

## 4. Materials and Methods

### 4.1. Plant Materials

*Phyllostachys edulis* seeds were collected from Guilin, Guangxi, China. After being sterilized and vernalized for three days at 4 °C, the seeds were put on filter paper and allowed to germinate in a climate room in the dark. Following a week of germination, the seedlings were moved to Hoagland nutritional solution and raised in an intelligent greenhouse with an average temperature of 23 °C and 70% humidity for about a month. The seedlings were given a 16 h light and 8 h dark photoperiod. Subsequent studies were conducted using the bamboo seedlings that were hydroponically cultivated.

### 4.2. Identification of the PeCHS Gene Family

The hidden Markov model (HMM) profiles of CHS (PF00195 and PF02797) were sourced from the Pfam database [75] (http://pfam.xfam.org/) and employed as seed models to query the local bamboo protein database utilizing HMMER3 [76] (http://hmmer.janelia.org/) with an E-value cutoff of ≤10^−20^. Redundant genes were eliminated, yielding an initial collection of CHS candidate sequences. SMART (http://smart.embl-heidelberg.de/) and the Pfam database were employed to validate the precision of the original screening by excluding sequences devoid of entire CHS domains. The officially verified PeCHS members were renamed accordingly.

### 4.3. Analysis of Physicochemical Properties

The online program Plant-mPLoc was utilized to predict subcellular localization. The physicochemical properties of each protein sequence, such as number of amino acids, molecular weight (MW), isoelectric point (pI), instability index, and grand average of hydropathicity (GRAVY), were evaluated using TBtools software v2.148.

### 4.4. Phylogenetic Tree Construction

CHS sequences from *Arabidopsis thaliana* and *Oryza sativa* were obtained using HMMER3 from their local protein databases and combined with the CHS sequences from *Phyllostachys edulis*. A phylogenetic tree was generated utilizing MEGA 7.0 through the Neighbor-Joining (NJ) method [77]. An intraspecies phylogenetic tree was also generated for CHS protein sequences in *Phyllostachys edulis*.

### 4.5. Collinearity Analysis

The 17 PeCHS protein sequences were aligned using BLASTP (E-value ≤ 10^−20^). Gene duplication events and collinearity relationships among the CHS proteins were identified using MCScanX [78]. The results were visualized with TBtools [79].

### 4.6. Gene Structure, Motif Composition, and Promoter Element Analysis

The intron–exon distribution of *PeCHS* genes was established using the GFF annotation file of *Phyllostachys edulis* genome. The conserved motifs of CHS proteins were examined utilizing the MEME online tool [80]. Cis-acting elements in the 2000 bp promoter region upstream of each gene’s transcription start site were discovered with PlantCARE. The conclusive outcomes were illustrated utilizing the TBtools program [81].

### 4.7. Prediction of Protein Tertiary Structure

The SWISS-MODEL web platform (https://swissmodel.expasy.org/) was used to estimate the protein’s tertiary structure. Using Discovery Studio, the final model was improved and shown.

### 4.8. Gene Expression Analysis Based on RNA-Seq Data

Gene expression datasets of roots, rhizomes, panicles, and leaves of Moso bamboo were acquired from the EMBL database (PRJEB2956). Transcriptome data of shoot tissues at 0.5, 1, 2, 3, 5, 6, and 7 m were downloaded from the NCBI-SRA database (PRJNA414226). Additionally, for the study of salinity and drought stress, seedling root tissues were treated with 100 mM gibberellin (GA), 100 mM naphthalene acetic acid (NAA), 1 mM salicylic acid (SA), 1 μM abscisic acid (ABA), 200 mM NaCl, and 25% PEG6000, and samples were taken 4 h after treatment. The transcriptome data of these treated root tissues were obtained from the NCBI-SRA database (PRJNA413166 and PRJNA715101). Additionally, RNA-Seq data for various plant tissues of *Phyllostachys edulis* (roots, rhizomes, panicles, and leaves) were downloaded from EMBL (https://www.embl.org/) with accession number PRJEB2956. The expression abundance of *PeCHS* genes was calculated in transcripts per million reads (TPM) [82]. The TPM values were log2 ^(TPM+1)^ transformed, and a heatmap of gene expression was generated using TBtools [83].

### 4.9. GO Enrichment Analysis

The Gene Ontology (GO) annotations of *CHS* genes were assigned using GOATOOLS (https://github.com/tanghaibao/GOatools). To find biological functions that were highly enriched in *Phyllostachys edulis CHS* genes when compared to the entire GO database, Fisher’s exact test was utilized. When the adjusted *p*-value (p. adjust) was less than 0.05, GO functions were deemed highly enriched. False discovery rate (FDR) correction was used to reduce false positives.

### 4.10. Construction of the PeCHS Gene Regulatory Network

The PlantPAN database was used to find putative transcription factor binding clusters in each gene’s 1000 bp upstream region. To find transcription factor binding sites, binding motifs in the promoter regions of the *CHS* gene were examined. Transcription factors were renamed in light of the findings (Supplementary Table S1). For motif identification, the FIMO web tool (https://meme-suite.org/tools/fimo) [84] and the JASPAR database (http://jaspar.genereg.net/) [85,86] were used. Lastly, the Omicshare platform (https://www.omicshare.com) was used to create a dynamic correlation heatmap that shows the link between *PeCHS* genes and their upstream transcription factor genes [87].

### 4.11. Subcellular Localization of PeCHS14 and PeCHS15

To create novel expression vectors, specific primers for the *PeCHS14* and *PeCHS15* genes were created, and the genes were fused with green fluorescent protein (GFP) using seamless cloning technology. Following their introduction into *Agrobacterium tumefaciens* strain *GV3101* [88], the properly cloned recombinant plasmids underwent a transformation into tobacco leaves. After being exposed to darkness for 24 h, the plants were then exposed to light for another 24 to 72 h. After preparing leaf sections, a laser confocal microscope (Olympus, Tokyo, Japan) was used to observe the localization of gene expression. Red fluorescent protein (RFP) and histone H2B fusion served as a nuclear marker.

### 4.12. QRT-PCR Experiment

To investigate the expression patterns of *PeCHS* gene family under hormone treatments at different time intervals, hydroponic seedlings approximately one month old, with similar size and growth, were selected as experimental materials. For hormone treatments, under long-day conditions with illumination (8 h of darkness), seedling leaves were sprayed with 100 μM ABA or GA solutions at 0 h, 3 h, 6 h, 12 h and 24 h. Leaves samples were collected post-treatment, with 0 h untreated samples as controls. Three biological replicates were collected randomly. The samples were promptly frozen in liquid nitrogen and maintained at −80 °C for further analyses [89]. RNA was extracted from each sample utilizing the FastPure Plant Total RNA Extraction Kit (Vazyme, Nanjing, China). First-strand cDNA was synthesized utilizing the HiScript^®^ III 1st Strand cDNA Synthesis Kit (+gDNA wiper) (Vazyme, China), which eliminates genomic DNA contamination via the gDNA wiper step.

Particular primers for qRT-PCR were designed utilizing the selected gene sequences with Beacon Designer 7.0, as detailed in Supplementary Table S2. Quantitative reverse transcription polymerase chain reaction (qRT-PCR) was performed utilizing the CFX-96 Real-Time System (Bio-Rad, Hercules, CA, USA) in accordance with the Ex Taq II (TaKaRa) protocol. Each sample underwent analysis with four technical replicates. The PCR protocol consisted of an initial denaturation at 95 °C for 5 min, followed by 38 cycles of denaturation at 95 °C for 15 s and annealing at 55 °C for 15 s. A melting curve ranging from 65 °C to 95 °C was produced to confirm specificity [90]. The internal control utilized was the reference gene *PeNTB*. Gene expression levels were quantified utilizing the 2^−ΔΔCT^ method. Data analysis and bar graph plotting were conducted utilizing GraphPad.

## 5. Conclusions

In this study, 17 *CHS* gene family members in *Phyllostachys edulis* were identified and comprehensively analyzed, including their physicochemical properties, phylogenetic relationships, chromosomal localization, collinearity, gene structure, conserved domains, protein tertiary structure, *cis*-acting elements, RNA-Seq expression profiles, and GO functional enrichment. Through the integration of transcription factor regulatory network correlations and subcellular localization data, the evolutionary relationships, functions, and response patterns of the *PeCHS* gene family under abiotic stress, hormone treatments, and tissue specificity were preliminarily explored. Additionally, GA and ABA treatments were selected for qRT-PCR validation. The results indicated that *PeCHSs* are relatively evolutionarily conserved, and five family members (*PeCHS04*, *PeCHS06*, *PeCHS08*, *PeCHS14*, and *PeCHS15*) exhibited distinct response patterns to hormone treatments across different time intervals. This study lays a foundation for future investigations into the potential functions of *PeCHSs* and offers candidate genes for further research on *Phyllostachys edulis*’s responses to abiotic stresses and hormonal regulation.

## Figures and Tables

**Table 1:** Physicochemical properties of the PeCHS gene family proteins in *Phyllostachys edulis*.

Name	Gene ID	Number of Amino Acids	MW (KDa)	PI	Instability Index	GRAVY
*PeCHS01*	*PH02Gene38478.t1*	405	42.724	6.32	35.55	0.052
*PeCHS02*	*PH02Gene37360.t1*	390	42.303	7.08	43.45	−0.068
*PeCHS03*	*PH02Gene38613.t1*	391	42.172	5.7	34.46	0.001
*PeCHS04*	*PH02Gene38615.t1*	394	42.538	5.93	32.1	−0.004
*PeCHS05*	*PH02Gene49028.t1*	394	42.538	5.93	32.1	−0.004
*PeCHS06*	*PH02Gene50391.t1*	391	42.317	5.96	40.66	−0.017
*PeCHS07*	*PH02Gene30447.t1*	390	42.314	6.08	33.3	−0.066
*PeCHS08*	*PH02Gene43021.t1*	388	42.208	5.72	36.63	−0.038
*PeCHS09*	*PH02Gene13137.t1*	402	43.789	5.66	39.11	−0.055
*PeCHS10*	*PH02Gene24703.t1*	354	38.067	6.41	43.08	−0.096
*PeCHS11*	*PH02Gene35700.t1*	393	42.014	5.89	34.69	0.036
*PeCHS12*	*PH02Gene35703.t1*	309	32.996	6.83	32.85	0.079
*PeCHS13*	*PH02Gene11618.t1*	390	42.066	5.7	35.84	−0.031
*PeCHS14*	*PH02Gene44458.t1*	368	39.940	6.1	43.11	−0.036
*PeCHS15*	*PH02Gene01692.t1*	396	42.945	5.54	35.49	−0.096
*PeCHS16*	*PH02Gene43281.t1*	402	43.266	6.38	40.58	−0.069
*PeCHS17*	*PH02Gene48527.t1*	393	41.954	6.02	33.84	0.04

MW: molecular weight; PI: isoelectric point; GRAVY: grand average of hydropathicity.

## Data Availability

The data from this study are available in the article and accompanying Supplementary Materials.

## Supplementary Materials

The following supporting information can be downloaded at: https://www.mdpi.com/article/10.3390/plants14020161/s1.
